# Effect of pulsed field ablation on solid tumor cells and microenvironment

**DOI:** 10.3389/fonc.2022.899722

**Published:** 2022-08-23

**Authors:** Yujue Wang, Tian’an Jiang, Liting Xie, Huiyang Wang, Jing Zhao, Lei Xu, Chengyu Fang

**Affiliations:** ^1^ Department of Ultrasound Medicine, The First Affiliated Hospital, Zhejiang University School of Medicine, Hangzhou, China; ^2^ Key Laboratory of Pulsed Power Translational Medicine of Zhejiang Province, Hangzhou, China; ^3^ Zhejiang University Cancer Center, Hangzhou, China

**Keywords:** pulsed field ablation, irreversible electroporation, nanosecond pulsed electric fields, immunogenic cell death, microenvironment

## Abstract

Pulsed field ablation can increase membrane permeability and is an emerging non-thermal ablation. While ablating tumor tissues, electrical pulses not only act on the membrane structure of cells to cause irreversible electroporation, but also convert tumors into an immune active state, increase the permeability of microvessels, inhibit the proliferation of pathological blood vessels, and soften the extracellular matrix thereby inhibiting infiltrative tumor growth. Electrical pulses can alter the tumor microenvironment, making the inhibitory effect on the tumor not limited to short-term killing, but mobilizing the collective immune system to inhibit tumor growth and invasion together.

## Introduction

The cells demonstrate atypia as they go from normal to malignant cells, including pleomorphism, hyperchromasia, and an increase in mitotic figure. The tumor tissues also show atypia, that is, the arrangement of tumor cells becomes disordered and irregular. Changes in the tumor microenvironment have gotten a lot of attention in recent years when it comes to the occurrence and progression of cancer. The tumor microenvironment, including tumor chemical environment, immune cells, extracellular matrix (ECM), and tumor vascular system, is the tiny environment in which tumors live (1). The enhancement of tumor proliferation signal, the resistance of apoptosis, avoidance of immunity, and promotion of tumor microvascular formation are all related to the microenvironment (2).

As an emerging ablation technique, irreversible electroporation (IRE) has the advantages of good tissue selectivity, clear ablation limits, no influence of large vessel heat sink effect, short ablation time, and few postoperative complications (3). Pulsed field ablation is different from traditional thermal ablation techniques such as radiofrequency ablation, microwave ablation, high intensity focused ultrasound therapy, etc. It is a heat-independent ablation with a delayed release of transient high-voltage electrical pulses that cause damage to the membrane structure of cells within the target ablation foci. The release of electrical pulses to tissues or cells can cause reversible or irreversible perforation of cell membranes, and apoptosis can be observed. There are several ablation techniques that perforate cells by electrical pulses to induce apoptosis, which can be classified according to the characteristics of the pulsed electric field parameters: nanosecond pulsed electric fields (nsPEF) deliver electrical pulses with very short pulse widths (in the range of 10-300 ns) and strong field strengths (20-150 kV/cm), and all pores remain small. IRE’s pulse widths range from microseconds to milliseconds but its amplitudes are less than 10 kV/cm, causing a wide range of pore size variations (4, 5). High-frequency irreversible electroporation (H-FIRE) systems that split the ~100 μs monopolar pulse into a series of shorter duration ~1 μs alternating polarity pulses (6). There is also electrochemotherapy (ECT), which allows the uptake of drugs by reversible electroporation (2). Despite the different pulse parameters, IRE, nsPEF, and H-FIRE can all act through irreversible damage to the cell membrane (4–6).

Studies have shown the safety and efficacy of pulsed field ablation (7–9). Pulsed field ablation can form perforations in the membrane and induce a complex immune process that alters the local microenvironment of the tumor (10, 11). In this review, we summarize changes in tumor cells, immunogenic effects, vascularity, extracellular matrix, and chemical environment induced by electric pulses.

## Changes in tumor cells

### Cell signal pathway

After delivering high-voltage electric pulses to tumor cells, it kills them *via* a variety of mechanisms including cell membrane perforation, mitochondrial damage, reactive oxygen species (ROS), and DNA damage (4, 12, 13). Firstly, IRE, nsPEF, and H-FIRE all cause damage to cell membranes, resulting in osmotic imbalance and cell swelling (14, 15). And electrical pulses can also lead to DNA damage, but whether the direct effect or the indirect effect induced by apoptosis is not clear (16–18). ROS is also one of the mechanisms of damage. High levels of ROS were found after PEF treated melanoma cells (19). What needs to be emphasized is that mitochondrial damage is more studied in nsPEF, because nsPEF has shorter pulse width, increasing the possibility of causing damage to organelles, and nsPEF causes mitochondrial damage by the loss of mitochondrial membrane potential (14, 20). Thus, damage to cells through different mechanisms may lead to changes in cellular signaling pathways.

Some articles have focused on the effects of electrical pulses on cellular signaling pathways. According to one study, applying nsPEF to the human pancreatic carcinoma cell line (PANC-1) can change the protein expression of the Wnt/β-catenin signaling pathway, matrix metalloproteinases (MMP) family, and vascular endothelial growth factor (VEGF). The downstream signals of the Wnt/β-catenin signaling pathway, including hDPR1, β-catenin, and c-Myc, are dose-dependently decreased by nanosecond pulses (21). Wnt/β-Catenin has two pathways, the canonical pathway and the non-canonical, and the canonical pathway can lead to the transcription of target genes such as myc and cyclin D1, nanosecond pulses inhibit the transcription of target genes through this pathway, thereby inhibiting the proliferation of tumor cells (22). In addition to Wnt/β-catenin pathway, the expression of NF-κB pathway proteins including IKK-α, IKK-β, IκB-α, NF-κB p-65, and p-p65 is also significantly reduced (21). Not only that, the expression of proapoptotic lymphocytes/leukemia-2 (Bcl-2) family proteins (Bax, Bim, and BID) is promoted, and the expression of antiapoptotic Bcl-2 family proteins phosphorylated Bcl-2 protein (p-Bcl-2), Bcl-xL and myeloid leukemia-1 (Mcl-1) are inhibited (22, 23). The MMPs family and VEGF are also lower than those of the control group. Downgrading of MMPs and VEGF can inhibit tumor invasion and metastasis. It is explained in detail in “4. Vascularity, stroma and chemical environment “.

Sun S et al. performed IRE on human pancreatic cancer cell line AsPC-1 and BxPC-3 *in vitro* and found that IRE can trigger ROS-dependent apoptosis in pancreatic cancer through the PI3K/Akt pathway (11). Another study found that the gene expression of KRAS and EGFR pathway signaling molecules changed significantly after IRE treatment on pancreatic tumors. EGFR signaling was inhibited: (i) causing a decrease in AKT, NF-kB, and VEGF expression, which inhibited tumor growth and invasion, metastasis, etc. (ii) leading to the inhibition of JAK and STAT3, thus providing inhibition of G0 to G1 phase transformation and reducing tumor cell replication. While K-RAS was inhibited, MEK1/2, JNK, and ERK1/2 expression were down-regulated, thus inhibiting cell replication and proliferation. IRE significantly altered the cancer hallmarks and immunosuppressive biological pathways in the PDX pancreatic tumor model. And necrosis, regeneration/repair, and inflammatory signaling were significantly increased after IRE (23).

**Figure 1:**
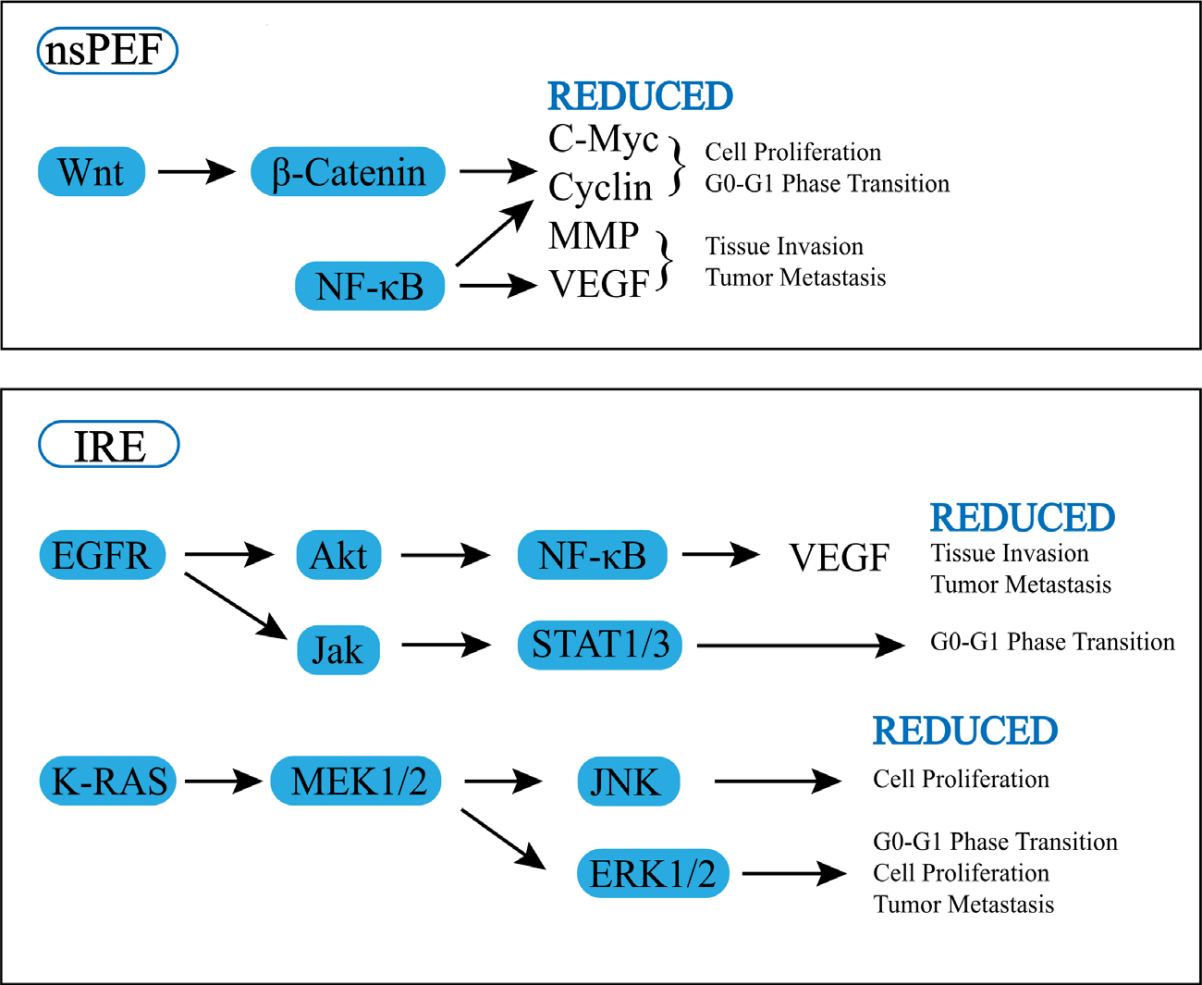
Effect of pulsed field ablation on cell signal pathway.

Wnt/β-Catenin, KRAS, EGFR, as well as downstream cellular pathways like MMP and VEGF were found to be downregulated after electrical pulses were applied to pancreatic cancer, and then cancer biology, including proliferation, cell death, invasion, and metastasis, all changed (**Figure 1**). Both IRE and nsPEF can exert anti-tumor effects by inhibiting cell replication, increasing the expression of pro-apoptotic proteins and suppressing the expression of antiapoptosis proteins, but there is not enough evidence to prove a significant difference between IRE and nsPEF in causing changes in cellular pathways.

### Cell death

Pulsed electric field ablation is known for its ability to cause apoptosis-a kind of programmed cell death. Because pro-apoptotic and anti-apoptotic factors regulate cell apoptosis, the increase in Bax, Bim, and BID and decrease in p-Bcl-2, Bcl-XL, and McL-1 after an electric pulse suggests that electroporation can promote cell apoptosis (18, 22, 24–26). Significantly increased cleavaged and active caspase 3, 7, and 9 were also detected after IRE (4, 21, 26–29), which are the markers of apoptosis. Cells exhibit the pathological characteristics of apoptosis after electrical impulses: nuclear pyknosis, nucleolysis, nuclear fragmentation, and apoptotic bodies were observed (21, 30–33).

However, during the delivery of electrical pulses, some heat will inevitably be generated. Tissues and cells exhibit distinct death features depending on their distance from the electrode needle. Generally speaking, the closer to the needle track, the easier it is to necrosis, the middle part shows irreversible electroporation, and the cells far away from the needle track are easy to form reversible perforation, which may be related to temperature, the closer the needle track is to the more heated the tissue, the more serious the thermal damage caused, which is characterized by zones of white coagulation (30, 34). The necrosis zone shows endoplasmic reticulum and nuclear membrane expansion and random DNA degradation (4, 16).

Pyroptosis and necroptosis belong to immunogenic cell death (ICD) that rely on the release of damage associated molecular patterns (DAMPs) to drive local immune responses. Pyroptosis forms intracellular inflammatory vesicles and activates caspase-1, gasdermin D (GSDMD) channels are formed on the cell surface and interleukin (IL)-1β, IL-18, and DAMP molecules are released from the cell *via* GSDMD pores, where they stimulate an immune response. Water and ion can also influx the cell from GSDMD, causing edema of the cell (4). Activation of caspase-1 and GSDMD was observed in rat liver tissue at 6 and 24 hours after electroporation, illustrating that IRE can cause pyroptosis (16). Necroptosis is initiated by the necrosome and activates the receptor interacting serine/threonine kinase 3 (RIPK3), which activates mixed lineage kinase domain-like pseudokinase (MLKL). Activated MLKL molecules aggregate and form pores in the cell membrane, allowing the release of DAMPs and the influx of water and ions, causing cellular edema and cell membrane disintegration, similar to the morphological manifestation of necrosis (4). Elevated RIP3 and MLKL were harvested after IRE, and cell morphology was observed with loss of the plasma membrane and release of organelles and chromatin, which is consistent with the morphology of necroptosis (21). Multiple modes of cell death may exist in the target area after electrical pulses, but they can change over time, and genetic analysis revealed that apoptosis was the predominant mode of cell death after H-FIRE (2000V, 100μs, bipolar pulses, a 2μs positive pulse, 5μs inter-pulse delay, 2;μs negative pulse, and a 5μs inter-pulse delay) was applied to the mouse 4T1 mammary tumor at 2 hours, while necrosis and pyroptosis were predominant by 24 hours (27). In addition, the mode of cell death can change with parameters, more energy may have greater thermal damage, more necrosis. Brock et al. conducted IRE on utilizing patient-derived xenograft (PDX) models, and found that apoptosis was evident at 500 V/cm but necrosis was more prominent at 2500 V/cm (23).

## Immune response

### DAMPs and immunity

Common DAMPs include the non-histone chromatin protein high mobility group box 1 (HMGB1), cell surface calcium reticulum protein (CRT), and other endoplasmic reticulum (ER) proteins, and adenosine triphosphate (ATP), which are associated with cell death. CD91, toll-like receptor 4 (TLR4), and The P2X7 receptor (P2RX7) are expressed by dendritic cells (DCs) and promote phagocytosis of dead cells, presentation of tumor antigens, and production of IL-1β, respectively (35). The release of DAMPs (ATP, calreticulin, nucleic acids and uric acid) increases with increasing pulse amplitude after IRE on cells *in vitro* (12, 29, 36–39) and causes massive immune cell aggregation in post-electroporation pancreatic cancer tissue *in vivo* (36) (**Table 1**). The release of DAMPs is related to the parameters of the pulses, at IRE (500-1500 V, 100 μs, 8-24 pulses) with increasing voltage, the release of DAMP increases (29), similarly, the number of DAMP releases is related to the number of pulses, after IRE (1000 V, 100 μs, 8/40/80 pulses), CRT, ATP, and HMGB1 were released most at 40 pulses and less at 8 and 80 pulses, suggesting that there may be a suitable number of pulses, neither too less nor too more, that would allow the most DAMP release, Go EJ et al. speculated that low pulses (<40) would not induce ICD and high pulses (>40) would lead to rapid cell death, thus limiting DAMP expression (38). Most of the studies about DAMP are *in vitro*, and the appropriate parameters, as well as the intensity-release dependence, may require further studies.

**Table 1:** Effects of pulsed field ablation on tumor microenvironment.

Factors	Intervention		Parameters				Mode of action	*In vitro* or *in vivo*	Type of tumor
		V	EFS	PW	PRF	N			
DAMP									
1.CRT	IRE & RE	1000 1000 1000	– – –	100 100 100	– – –	80 40 8	24h: Increased by about 6.1 times. 24h: Increased by about 30 times. 24h: Increased by about 6.9 times	*In vitro*	The Lewis lung carcinoma (LLC, CRL-1642) (38)
2.ATP	IRE & RE	200	–	100	1	20	Within 30min: No significant difference.	*In vitro*	KRAS* (36)
		960 200 960	– – –	100 100 100	1 1 1	20 20 20	Within 30min: Increased Within 30min: Increased slightly Within 30min: Increased	*In vitro*	KRAS* (36) B16F10 (36) B16F10 (36)
		1000	–	100	–	80 40 8	24h: Increased by about 1.6 times. 24h: Increased by about 8.7 times. 24h: Increased by about 5.4 times.	*In vitro*	The Lewis lung carcinoma (LLC, CRL-1642) (38)
		500 1000	– –	100 100	1 1	20 20	Increased	*In vitro*	KPC (37)
	nsPEF	– –	7000 7000	0.2 0.2	10 10	– –	No significant difference (CT26) Increased (EL-4)	*In vitro*	EL-4 lymphoma; CT26 colon carcinoma cells (39)
3. HMGB1	IRE & RE	200 960	– –	100 100	1 1	20 20	Within 30min: No significant difference at 200V, increased at 960V.	*In vitro*	KRAS* (36)
		200 960	– –	100 100	1 1	20 20	Within 30min: No significant difference at 200V, increased at 960V.	*In vitro*	B16F10 (36)
		500–1500	–	100	–	8 16 24	24h: Increased in a strength-dependent manner.	*In vitro*	Panc-1, Bxpc-3, Pan02 (29)
		1000	–	100	–	8 40 80	24h: Increased by about 7.3 times. 24h: Increased by about 12.3 times. 24h: No increase.	*In vitro*	The Lewis lung carcinoma (38)
	nsPEF	– –	7000 7000	0.2 0.2	10 10	– –	Increased Increased	*In vitro*	EL-4 lymphoma; CT26 colon carcinoma cells (39)
4.HSP70	IRE & RE	500–1500	–	100	–	24 16 8	24h: Increased in a strength-dependent manner	*In vitro*	Panc-1, Bxpc-3, Pan02 (29)
5.Calreticulin	IRE & RE	500–1500	–	100	–	24 16 8	24h: Increased in a strength-dependent manner	*In vitro*	Panc-1, Bxpc-3, Pan02 (29)
Phagocytes									
1.Macrophages	IRE	1000	–	100	1	80	Day 7: M1 polarized and Increased in a strength-dependent manner Day 7: M2 decreased	*In vivo*	PC (29)
2.DC	IRE	1200	–	100	1	99	Day 9: No significant difference.	*In vivo*	PC (36)
3.NK	IRE	–	–	–	–	–	Day 3: decreased Day 7: increased	*In vivo*	PC (37)
	nsPEF	20000	–	0.3	4	1000	Day 8: increased	*In vivo*	HCC (56)
**Cytokines and complements**									
IL-1a	IRE	3000	–	70	–	90	2 h: increased	*In vivo*	HCC (51)
IL-1b	IRE	3000	–	70	–	90	2 h: increased	*In vivo*	HCC (51)
	nsPEF	30000	–	0.3	–	400	Day 7: increased	*In vivo*	PC (53)
IL-2	IRE	3000	–	70	–	90	2 h: increased	*In vivo*	HCC (51)
		–	–	–	–	–	Day 7: increased (more than Day 3 and preOP)	*In vivo*	PC (50)
	nsPEF	20000	–	0.3	4	1000	Day 8: increased	*In vivo*	HCC (56)
IL-5	nsPEF	20000	–	0.3	4	1000	Day 8: increased	*In vivo*	HCC (56)
IL-6	IRE	–	–	–	–	–	Day 3: increased Day 7: decreased	*In vivo*	PC (50)
	nsPEF	20000	–	0.3	4	1000	Day 8: increased	*In vivo*	HCC (56)
		30000	–	0.3	–	400	Day 3: decreased	*In vivo*	PC (53)
IL-10	IRE	–	–	–	–	–	Day 3: increased Day 7: decreased	*In vivo*	PC (50)
		3000	–	70	–	90	2 h: increased Day 2: increased dramatically	*In vivo*	HCC (51)
	nsPEF	20000	–	0.3	4	1000	Day 8: increased	*In vivo*	HCC (56)
IL-12	IRE	3000	–	70	–	90	2 h: increased Day 2: increased dramatically	*In vivo*	HCC (51)
IL-17A	nsPEF	20000	–	0.3	4	1000	Day 8: increased	*In vivo*	HCC (56)
IL-17F	nsPEF	20000	–	0.3	4	1000	Day 8: increased	*In vivo*	HCC (56)
IL-21	nsPEF	20000	–	0.3	4	1000	Day 8: increased	*In vivo*	HCC (56)
IL-22	nsPEF	20000	–	0.3	4	1000	Day 8: increased	*In vivo*	HCC (56)
IFN-γ	IRE	–	–	–	–	–	No significant difference	*In vivo*	PC (50)
		3000	–	70	–	90	2 h: increased Day 2: increased dramatically	*In vivo*	HCC (51)
	nsPEF	20000	–	0.3	4	1000	Day 8: increased	*In vivo*	HCC (56)
TNF-α	IRE	3000	–	70	–	90	2 h: increased Day 2: increased dramatically	*In vivo*	HCC (51)
	nsPEF	30000	–	0.3	–	400	Day 7: increased	*In vivo*	PC (53)
		20000	–	0.3	4	1000	Day 8: increased	*In vivo*	HCC (56)
GM-CSF	IRE	3000	–	70	–	90	2 h: increased Day 2: increased dramatically	*In vivo*	HCC (51)
C3	IRE	–	–	–	–	–	Day 3: decreased Day 7: increased	*In vivo*	PC (50)
C4	IRE	–	–	–	–	–	Day 3: decreased Day 7: increased	*In vivo*	PC (50)
**Immune-suppressive cells**									
1.Treg	IRE	–	–	–	–	–	Day 3: increased Day 7: decreased	*In vivo*	PC (50)
		–	1500	90	–	–	Week 2: decreased	*In vivo*	PC (46)
		1200	–	100	1	99	Day 9: No significant difference	*In vivo*	PC (36)
	nsPEF	–	30000	0.3	–	400	Day 3: slightly increased Day 7: significantly decreased	*In vivo*	PC (53)
		–	30000	0.1	1	200	Day 4: decreased	*In vivo*	Malignant melanoma (67)
	H-FIRE	–	2500	100	–	–	Day 2: increased	*In vivo*	4T1 mammary tumor (27)
2.TAM	H-FIRE	–	2500	100	–	–	Day 2: decreased	*In vivo*	4T1 mammary tumor (27)
3.MDSC	IRE	–	1500	90	–	–	Day 14: eMDSC decreased	*In vivo*	PC (46)
	nsPEF	–	30000	0.3	–	400	Day 3&7: nMDSC & mMDSC decreased	*In vivo*	PC (53)
		–	30000	0.1	1	200	Day 4: decreased	*In vivo*	Malignant melanoma (67)
	H-FIRE	–	2500	100	–	–	Day 2: pMDSC decreased	*In vivo*	4T1 mammary tumor (27)
4.TAN	H-FIRE	–	2500	100	–	–	Day 2: decreased	*In vivo*	4T1 mammary tumor (27)
Adaptive immunity									
CD 4+ T cell	IRE	–	–	–	–	–	Week 2: increased	*In vivo*	PC (38)
		1200	–	100	1	99	Day 9: No significant difference	*In vivo*	PC (36)
		–	–	–	–	–	Day 3: decreased Day 7: increased	*In vivo*	PC (50)
	nsPEF	–	20000	300	4	1000	Day 8: increased	*In vivo*	HCC (56)
CD 8+ T cell	IRE	1200	–	100	1	99	Day 9: increased	*In vivo*	PC (36)
		–	–	–	–	–	Day 3: decreased Day 7: increased	*In vivo*	PC (50)
		1000	–	100	–	90	increased	*In vivo*	HCC (70)
	nsPEF	–	20000	300	4	1000	Day 8: increased	*In vivo*	HCC (56)
B cell	IRE	1200	–	100	1	99	Day 9: No significant difference	*In vivo*	PC (36)
	nsPEF	–	20000	300	4	1000	Day 8: increased	*In vivo*	HCC (56)
IgA	IRE	–	–	–	–	–	Day 3&7: No significant difference	*In vivo*	PC (50)
IgG	IRE	–	–	–	–	–	Day 3: decreased Day 7: increased	*In vivo*	PC (50)
IgM	IRE	–	–	–	–	–	Day 3&7: No significant difference	*In vivo*	PC (50)
**Vasculature, extracellular matrix, and chemical environment**									
VEGF	nsPEF	–	20000	0.1	–	100	1h: decrease	*In vivo*	HCC (21)
CD31	IRE	1000	–	100	1	80	Day 7: increased	*In vivo*	PC (46)
		1200	–	100	1	99	Day 4: transient increase Day 6: decreased	*In vivo*	PC (36)
CD34	nsPEF	–	20000	0.1	–	100	1h: decrease	*In vivo*	Hep-3B HCC (21)
FITC-conjugated dextran	IRE	1200	–	100	1	99	Day 4: increased Day 6: decrease, but still higher than that of untreated tumors	*In vivo*	PC (36)
FAPα	IRE	1200	–	100	1	99	Day 4: decreased Day 6: rebounded back	*In vivo*	PC (36)
HABP1	IRE	1200	–	100	1	99	Day 6: decreased	*In vivo*	PC (36)
	nsPEF	1000	–	100	1	80	Day 3: decreased Day 7: decreased	*In vivo*	PC (53)
LOX	IRE	1000	–	100	1	80	Day 3: decreased Day 7: decreased	*In vivo*	PC (46)
		1200	–	100	1	99	Day 6: decreased	*In vivo*	PC (36)
α- SMA	IRE	1200	–	100	1	99	Day 6: No significant difference	*In vivo*	PC (36)
	nsPEF	1000	–	100	1	80	Day 3&7: No significant difference	*In vivo*	PC (53)
MMP	nsPEF		20000–60000	100	–	100	1 h: decrease	*In vivo*	HCC (21)
CA-IX	IRE	1200	–	100	1	99	Day 6: decreased	*In vivo*	PC (36)
HIF-1α	IRE	1200	–	100	1	99	Day 6: decreased	*In vivo*	PC (36)

V, Voltage (V); EFS, electric field intensity (V/cm); PW, Pulse width (µs); PRF, Pulse repetition frequency (Hz); N, Number of pulses; min, minutes; h, hour; HCC, hepatic cancer; PC, pancreatic cancer; RE, reversible electroporation; IRE, irreversible elecrtroporation; nsPEF, nanosecond pulsed electric fields; H-FIRE, High-frequency irreversible electroporation.

(i) CRT is the most abundant in the endoplasmic reticulum. After activation of ICD-related signaling pathways, it transfers from the endoplasmic reticulum to the cell membrane surface and can interact with transmembrane receptors including CD49, CD69, CD91 (also known as the low density lipoprotein (LDL) receptor-related protein-1 (LRP1)), and integrins. The most important is the CD91 molecule. CRT releases effective phagocytic signals to CD91-positive cells (mainly macrophages and DCs) and causes the production of pro-inflammatory cytokines (including IL-6 and TNF-α) (35, 40). (ii)In addition to participating in purinergic neurotransmission, ATP released from damaged cells can bind to the P2Y2 receptor of macrophages, promoting the infiltration of macrophages in tumor sites, and can also bind to the P2RX7 of DC cells, leading to DC maturation and release of IL-1β. (iii) HMGB1 can bind to protein toll-like receptor 4 (TLR-4) and receptor for advanced glycation end products (RAGE) to activate monocytes/macrophages. HMGB1 can also upregulate costimulatory molecules and major histocompatibility complex (MHC) class II to transfer immature DC to mature DC (35, 41, 42). HMGB1 stimulates neutrophils and monocytes, enabling these cells to adhere to activated vascular endothelium and migrate to inflamed tissues (43).

Electrical pulse stimulation triggers the release of DAMPs, which acts as a “find me” signal, enhances tumor immunogenicity and subsequently induces antigen-presenting cells (APC) activation. These signals enhance the ability of APC to phagocytose, process, and present tumor-derived antigens to T cells, thereby facilitating the induction of tumor-specific adaptive immunity. So, the level of these DAMPs and cells increases after pulsed electric field (29, 36–39).

### Innate immune

#### Phagocytes

There are many phagocytic cells in the body, and the first one worth mentioning is macrophages. Macrophages have multiple functions: phagocytosis of dead cells and debris; acting as APC to process antigens and participate in adaptive immunity through MHC molecules; production and secretion of cytokines, including IL-1, IL-6, TNF-α, etc (44).

Polarized macrophages mainly exist in two distinct subsets: M1 and M2. The secreted cytokines are the key feature to distinguish the two: M1 type can secrete IL-6, IL-12 and tumor necrosis factor (TNF), M2 type can secrete IL-10, IL-1 receptor antagonist (IL-1ra), and the type II IL-1 decoy receptor. Type M1 is an effective inflammatory effector cell that can produce a large number of cytokines and kill tumor cells through the production of ROS. Type M2 is more inclined to promote angiogenesis and promote fibrosis to remodel and repair tissues (44, 45). Tumor-associated macrophages (TAM) have a phenotype and function similar to M2 macrophages, which reduce the killing of tumor cells by cytotoxic T cells and NK cells (45). Tumor cells secrete chemokine (C-C motif) ligand 2 (CCL-2) (lung tumors, breast cancer, cervical cancer, ovarian cancer, etc.) to cause the accumulation of macrophages. Low levels of CCL-2 promote tumor growth, and high levels of CCL-2 cause a large number of macrophages to accumulate and tumor destruction (45). After pancreatic ductal cell adenocarcinoma (PDAC) undergoes electroporation, the expression of CD16/32 in macrophages (a hallmark of M1 macrophages) increases and changes from a rod shape to a round shape, indicating that the formation of irreversible electroporation can induce M1 macrophages polarization of cells. In addition, positive-feedback release or expression of HMGB1 and RAGE in macrophages *via* the MAPK-ERK pathway promoted M1 macrophage polarization (29, 38), and M1/M2 ratio tends to increase in a strength-dependent manner (29). In addition to the MAPK - ERK pathway, a stimulator of interferon genes (STING) signaling is involved in the activation and repolarization of macrophages, one study found that this macrophage repolarization was most pronounced when tumors were treated with a combination of IRE and STING agonist (38).

After the electric pulse acts on the tissue, in addition to macrophages, the ablation zone also found the accumulation and activation of neutrophils, DC cells, and NK cells (**Table 1**). Like macrophages, these phagocytes can kill perforated cells (10, 36, 37, 46, 47).

Immature DC cells highly express TLRs, opsonizing receptors, etc. After receiving the DAMPs signal released by the perforated cells, the low-expressed MHC class II molecules and costimulatory molecules are activated to become mature DC cells, which effectively present antigens in adaptive immunity (48). After electric pulse treatment of mouse KRAS* cells *in vitro*, the CD40, MHC-II, chemokine receptor (CCR) 7, and CD86 surface markers of DC cells increased relatively, which suggested the activation of DC cells (36). Combining IRE and DC vaccines for mouse pancreatic cancer, it can be found that IRE can overcome the immunosuppressive environment of pancreatic cancer, thereby enhancing the effect of DC vaccination (37).

NK cells can be defined into two subsets according to the levels of CD56 and CD 16: CD56^hi^ CD16^±^ and CD56^lo^ CD16^hi^, the former promoting the inflammatory response by releasing cytokines and the latter killing cells by perforin and granzyme (49). IRE can increase the concentration of mouse NK1.1 cells in the blood and tumor accumulation in animal experiments (37), and it can also cause an increase in peripheral blood NK cells in humans (50). NK cell therapy can also increase the killing effect on tumor cells. The combination of IRE ablation and NK cells can have a synergistic therapeutic effect on stage IV hepatocellular carcinoma. The combined treatment group’s IL-2, tumor necrosis factor (TNF), and interferon (IFN) levels are higher in both groups than in the single treatment group. Synergistic treatment of liver cancer with IRE and NK also increases the levels of lymphocytes and Th1-type cytokine decreases the expression of alpha-fetoprotein and increases the survival time of patients (49). So, increasing NK cells will inhibit tumor growth, and electrical pulses can have a synergistic effect with NK cell therapy.

#### Cytokines

Chen X found IL-1a, IL-1ra, IL-1b, IL-2, IL-6, IL-8, and IL-18 levels are significantly higher 2 hours after IRE ablation. IL-4, IL-10, IL-12, TNF-a, IFN-r, granulocyte-macrophage colony-stimulating factor (GM-CSF) increased dramatically 2 days after ablation (51). Most of these cytokines can activate cytotoxic immunity, including IL-2, IL-4, IL-5, IL-6, IL-7, IL-10, IL-12, and IL-15. IL-16 and IL-17 also facilitate cellular immunity (52). And Chen X’s result indicated that changes the abnormal drifted Th2 in HCC back to Th1 status (51). Zhao et al. found that after seven days the TNF-α and IL-1β levels in blood were increased, while IL-6 levels were decreased (53). IFN-γ stimulates antigen presentation and cytokine production by monocyte, and also stimulates monocyte adhesion, phagocytosis, and other effector functions. One of the most important biological activities of IL-1 is its ability to activate T lymphocytes by enhancing IL-2 production and IL-2 receptor expression. IL-6 is mainly produced by monocytes and mediates T cell activation, growth, and differentiation (52). IL-10 is a compound with both immunosuppressive and anti-angiogenic functions and is a direct inhibitor of Th1 function (54, 55). Yimingjiang et al. found significantly higher IL-10 in tumor-bearing mice after nanosecond pulses than in controls (56), while He et al. found that after IRE, IL-10 levels in pancreatic cancer increased on day 3 and decreased on day 7 (46). The immunosuppressive effect of IL-10, the function of recruitment to Treg makes IL-10 seem to promote tumor growth, while the changes in IL-10 levels after electrical pulses vary from experiment to experiment and need to be further verified (46, 51, 52, 56).

Thus, electrical pulses can activate phagocytosis, adhesion phagocytosis, activation of T lymphocytes, and induction of cytotoxic T lymphocyte (CTL) direct killer cells for immune response to post-perforation cells by triggering the secretion of pro-inflammatory cytokines *in vivo*.

### Immune-suppressive cells

A large number of immunosuppressive cells are present in tumors, including T regulatory cells (Tregs), tumor-associated macrophages (TAMs), cancer-associated fibroblasts (CAFs), and myeloid-derived suppressor cells (MDSCs), and the upregulation of these cell types in tumors depends on the reciprocal signaling between these cells and tumor cells.

The production of Treg (usually CD4+CD25^+^Foxp3^+^ T cells) depends mainly on transforming growth factor-β (TGF-β) and IL-2, which negatively regulate immunity and can produce TGF-β and IL-10 to suppress immune responses (55, 57, 58). And Tregs’ infiltration is negatively correlated with median survival OS in many patients with solid tumors (59). Tregs can effectively suppress effector T lymphocytes and can inhibit the function of B, NK, dendritic cells, and macrophages through different mechanisms (58, 60).

TAM has an M2 macrophage-like phenotype and promotes tumor progression through several mechanisms: secretion of VEGF, which promotes tumor angiogenesis; promotion of tumor invasion mainly through the release of metalloproteinases, matrix remodeling enzymes, and chemotactic growth factors from the environment; and suppression of innate immune responses (61).

There are mainly two types of MDSC: polymorphonuclear MDSC (P-MDSC) which resemble neutrophils morphologically and phenotypically, and monocyte MDSC (M-MDSC) which resemble monocytes. MDSC has potent immunosuppressive activity through multiple pathways: promoting Tregs’ production and promoting fibroblast differentiation into cancer-associated fibroblasts (CAF) depleting L-arginine eliminates key trophic factors required for T cell proliferation, nitrates chemokines and blocks CD8+ T cells from entering the tumor, and produces immunosuppressive cytokines such as IL-10 and TGF-β (61, 62).

Unlike normal myofibroblasts, CAF does not undergo apoptosis and can release various cytokines and MMPs to hydrolyze extracellular matrix, stimulate angiogenesis and promote tumor growth and invasion (63). (As described in 4. Vasculature, extracellular matrix, and chemical environment).

Reduction of systemic Tregs in locally advanced pancreatic cancer (LAPC) patients 2 weeks after IRE was found in clinical trials (64). However, a transient increase in Tregs on day three followed by a decrease on day seven was found in the clinical trial by He C (46). Similar results were also found in Harshul et al.’s study, where LAPC patients could have a procedure-mediated Treg attenuation between the third and fifth day after IRE (65). A reduction in Li^-^ CD^33+^ HLA^-^DR^-^ early myeloid-derived suppressor cells (eMDSC) was observed 2 weeks after IRE treatment (64). IRE combined with OX40 agonist induced a significant reduction in MDSC in primary and distant tumors (66). H-FIRE resulted in a reduction of MDSCs and TAMs in the tumor microenvironment of mammary carcinoma in mice 2 days after procedure (27). NsPEF treated with C57 malignant melanoma reduced Treg cells from 4.3% to 2.4% and MDSC by 39.0% to 19.7%, which was observed 4 days later (67). NsPEF can act on mice with pancreatic cancer after 3 days postoperative, 7 days postoperative decreased the percentage of nMDSCs and mMDSCs in the spleen, although Tregs slightly increased at 3 days postoperatively, but significantly decreased at 7 days postoperative (53), indicates that the immunosuppressed state can be reversed in this period of time, which would facilitate the combination with immunotherapy.

Therefore, electrical pulses can inhibit the proliferation of tumor-associated immune cells in the tumor microenvironment and promote anti-tumor responses to create an immune environment conducive to tumor suppression. However, the reversion of immunosuppression after IRE or nsPEF is time-dependent and this may start after day 3, but a longer and more subtle follow-up is needed to determine the time window for combination with immunotherapy.

### Adaptive immunity

Adaptive immunity is achieved through regulated interactions between APC and T and B cells. Circulating antigens or APC-treated antigens are presented to T and B cells, eliciting cellular and humoral immunity, respectively. The largest T cell population in the body is the CD4+αβ T cell receptor (TCR) population. Most of these cells have a helper function and are called helper T (Th) cells, which produce many cytokines. CTL is a type of CD8+ T cells that kill target host cells through a contact-dependent mechanism: increased expression of FasL on CTL binds to Fas receptors in target tissues, participates in apoptosis, and acts on target cells by releasing substances such as perforin and granzyme. Adaptive humoral immunity is mediated by antibodies produced by plasma cells (55).

Several studies have found that electrical pulses acting on cells induce increased circulation and ablation foci of CD8+ T cells (24, 37, 46, 64, 68–70), and some experiments have found elevated CD4+ levels (38, 46, 55, 56), however, some studies has also shown no significant increase in CD4+ levels (10, 23) (**Table 1**). Zhao et al. found an increased CD8+ T cells and CD4+ T after nanosecond pulses acting on pancreatic cancer in mice, and a significantly higher CD8/CD3 ratio in tumors compared to controls (53). He et al. found an increase of effector CD8+ T cells, effector CD4+ T cells, and memory T cells at 7 days after IRE, despite decrease at day 3, so it can effectively induce the activation of T cells over a period of time, and the experiment also found that IRE can inhibit the growth of potential tumors through the distant effect (50). However, Dai et al. implied that IRE treatment significantly inhibited HCC growth by more CD8+ T and dendritic cells, but not CD4+ T or B cells infiltrating into the peri-ablative region. CD8+ depleted T cells induced local tumor regeneration and distant metastasis after IRE (10). Most of the IRE or nsPEF studies have activated the proliferation of CD8+T, but the proliferation of CD4+T is not obvious in some studies, revealing that CD8+ T-mediated cellular immunity plays a great role in electric pulses induced immunity. Effective T cell initiation requires several events, including: release of endogenous antigens from cancer cells, release of “danger signals” from damaged cells, processing of cancer antigens, antigens presented to naive T cells by APC, activation and proliferation of cancer-specific cytotoxic T cells (55, 69, 71). The current results suggest that pulsed electric field can promote cellular immunity through these sessions: 1) induce immunogenic death, resulting in the massive release of DAMP (29, 36, 38, 39); 2) Proliferation and activation of antigen presenting cells (29, 36, 38); 3) Activation, proliferation and function of cancer-specific cytotoxic T cells (36, 64, 66, 67, 70). In addition, Shao et al. compared IRE, thermal therapy (Heat), cryosurgery (Cryo) *in vitro*, and found that IRE can cause more protein release than other ablation. Although the released protein has 40% denatureation, T cell proliferation is still 2-3 times higher than Cryo (69). IRE induces OX40 expression in CD8+ T cells *in vivo*, and OX40 acts as a co-stimulatory molecule to increase T cell expansion and cytokine secretion (66). The combination of IRE and TLR 3/9 agonists and PD-1 blockade can effectively reverse the depletion of intratumoral CD8+T and enhance local immunity against tumors (72). Brandon et al. made a deeper exploration by combining anti-T-lymphocyte-associated protein-4 (anti-CTLA-4) therapy prior with IRE on prostate cancer to promote neoantigen-specific T-cell responses, resulting in increased numbers of splenic systemic SPAS-1+ T cells concentrated in tumors and distant sites. Circulating memory CD8+ T cells, in addition to central memory (T_CM_) and effector memory (T_EM_), have tissue-resident memory (T_RM_). Endogenous SPAS-1 neoantigen-specific CD8+ T cells were increased in number and enriched in tumors following TRAMP-C2 tumor cell were attack and generated CD8+ T_RM_ cells in different tissues (68). In addition, Shi et al. treated hepatocellular carcinoma (HCC) with IRE in combination with an anti-PD-L1 monoclonal antibody and found enhanced off-target necrosis and inflammatory infiltration, with IRE significantly increasing the inflammatory infiltration index and increasing CD8+ T infiltration not only in target tissues but also in non-target tissues (untreated tumors) (70). Immunotherapy Combined IRE induced more CD8+ T proliferation and enrichment in tumors as well as other sites than immunotherapy alone, probably because: 1) IRE increased its immunogenicity: IRE caused immunogenic death of tumor tissues, massive release of DAMPs, causing activation of APCs and presentation to T cells, leading to tumor specific T-cell population expansion and enhanced systemic antitumor effects; 2) Reversal of the immune tolerant tumor microenvironment, with M1 macrophages polarizing CD4+ Th1 cell differentiation to enhance CD8+ T cell survival and tumor infiltration; 3) IRE-induced regulation of the tumor stroma, extracellular matrix, and/or vascular system may be another reason (21, 36, 46, 53, 68, 73).

## Vasculature, extracellular matrix, and chemical environment

### Vasculature

Several studies have demonstrated the protective effect of ablation foci on large vessels (9, 16, 74). For example, researchers followed 158 vessels with a mean distance of 2.3 ± 2.5 mm from the treatment area and found only 7 (4.4%) with abnormal vascular changes, including stenosis and thrombosis (9). However, the effect of IRE on microvessels is uncertain, and in some studies, microvessels remain histopathologically preserved in the area after ablation and the structure is still present (75), but can show microvascular distortion, occlusion, and thrombosis when observed under electron microscopy (32), and after disruption of vascular continuity there can be hemorrhagic necrosis with infiltration of surrounding neutrophils (76), and endothelial cells are damaged significantly. Thereafter, the disrupted vessel can be recognized by new endothelial cells derived from neighboring cells and/or circulating endothelial progenitor cells (32). Non-thermal irreversible electroporation can cause a decellularizing effect of the vessel at 3 days, the vessel skeleton survives while cells are shed, however, at 7 days this skeleton has endothelial ingrowth (74).

The changes of the microvasculature after IRE are: immediate congestion (75); necrosis of endothelial cells, hemorrhage, and peripheral inflammatory response (32, 76); and there can be regeneration of new vessels (32). It is worth mentioning that in Lv et al.’s theoretical study of the effect of perforation on tumor vasculature and normal vasculature, by establishing a multilayer dielectric model, explored that rich vascular smooth muscle cells (VSMCs) might have a protective effect on normal vasculature, thus demonstrated that electroporation may have a stronger destructive effect on tumor vasculature (77).

At the level of regulation of angiogenesis, tumor growth requires nutritional support from blood vessels, and angiogenesis is influenced by the expression of pro-angiogenic factors and anti-angiogenic factors; the VEGF family, composed of six growth factors (VEGFA-F), is essential for angiogenesis (78, 79), and angiopoietin 1-2 (Ang1-2) is independent of VEGF, while Ang-2 is mainly present in vascular expressed in remodeled tissues and in the hypoxic tumor microenvironment (80). VEGF can also exert inhibitory effects on DC cells and effector T cells in driving neoangiogenesis, as well as increase TAM infiltration and the expansion of Tregs and MDSCs (78, 81–84). However, due to the overexpression of pro-angiogenic factors and less in tumors, tumor vessels exhibit functional abnormalities with abnormal leakage, rapid growth, high tortuosity, and little perivascular pericytes and smooth muscle cells coverage (78, 79). A decrease in VEGF and CD34 proteins can be detected 1 hour after nanosecond pulse treatment of pancreatic cancer (21). He et al. also found increased expression of CD31 in tumor after IRE (53). In addition, nsPEFs and everolimus (The mammalian target of rapamycin (mTOR) inhibitor) synergistically inhibited angiogenesis by decreasing the expression of vascular endothelial growth factor (VEGF), VEGF receptor (VEGFR), and CD34 (85). In addition to inhibiting the expression of pathological proangiogenic factors, a study by Zhao et al. found a transient increase in CD31 calculated tumor microvascular density microvessel density (MVD) followed by a decrease four days after IRE treatment of pancreatic cancer and an increase in microvascular permeability determined by fluorescein isothiocynate (FITC)-bound dextran (73). Therefore, pulsed electric field can inhibit the growth of tumor pathological blood vessels and blood supply around the tumor, and also preserve the permeability of functional blood vessels to a certain extent, which is conducive to the infiltration of immune cells and factors.

### Extracellular matrix

In the tumor microenvironment, not only tumor cells proliferate rapidly, but also stromal deposition and remodeling as well as cancer cells and stromal cells increase, and CAFs form the main support structure of tumor tissues (1, 2). CAFs also promote cancer development by secreting growth-promoting factors such as TGF-β, stromal degrading enzymes and angiogenic factors such as MMP or VEGF, α smooth muscle actin (α-SMA) is a reliable biomarker for CAFs, and fibroblast activating protein α (FAP-α, seprase) is a surface glycoprotein that is selectively expressed on solid tumor fibroblasts. MMP hydrolyzes the extracellular matrix and its expression correlates with the aggressive phenotype of tumor cells and tumor progression (86).

Extracellular matrix and collagen structures can exist intact after IRE action because IRE acts on phospholipid bilayers (3, 74).

MMPs family proteins (MMP1, MMP2, MMP9, MMP11, MMP12, MMP14, and MMP21) are expressed at different levels of nsPEF intensity (21). In a study by Zhao et al. collagen matrix or αSMA+ CAFs were not affected by IRE, and FAP-α, hyaluronic acid (indicated by HABP1 expression levels) and lysyl oxidase (LOX, a marker of extracellular matrix stiffness) were decreased to varying degrees (36). Vasculature and collagen were still present in IRE-treated lung tissue 2 days after treatment and 28 days after a significant increase, indicating remodeling and regeneration of the mesenchyme, but decorin and heparan sulfate decreased after ablation (87).

Therefore, when electric pulses cause irreversible electroporation of cells, the presence of stromal and collagen structures can be observed histopathologically, but they can also microscopically modulate the cellular matrix and reduce the levels of CAFs and MMPs (**Table 1**). With the preservation of functional vessels and increased vascular permeability, softened extracellular matrix is beneficial to infiltration of inflammation and distant effects (16, 36, 53, 67).

### Improving hypoxia

Tumor vessels show characteristics of tortuous, twisted, and easily occluded, and the tumor presents a relatively hypoxic state due to the rapid proliferation of tumor cells and the increase of extracellular matrix leading to the increase of tumor tissue pressure. Hypoxia leads to the accumulation of hypoxia-inducible factor 1-α (HIF-1α), which promotes further tumor angiogenesis and suppresses T-cell function (2, 88). Moreover, hypoxia increases anaerobic enzymes and lactate accumulation further reduces T and NK cell activation (89). Reversal of intratumoral hypoxia effectively increases the infiltration of immune cells. The downregulation of HIF-1α and carbonic anhydrase 9 (CA-IX) and increased vascular permeability after IRE suggest that IRE may also increase the number and action of local T cells, NK cells by alleviating tumor hypoxia (36).

## Discussion

Compared with other local thermal techniques, pulsed electric field has several advantages in the regulation of the microenvironment: 1) It can protect the structure of large and medium vessels, and the elastic fibers and smooth muscle fibers in vessels can maintain the basic normal structure of vessels, with some damaged endothelial cells can be replaced (32, 74). 2) The protection of functional blood vessels makes sure the cell’s “eat me” signals be found and recognized by APC (10, 29, 37, 43). 3) APC presents antigens to activate immunity, and the retained blood vessels are more conducive to the infiltration of immune cells, which may reduce the occurrence of residual cancer (10, 29, 46). 4) Triggering a shift from the innate immunosuppressive microenvironment to the immune-promoted antitumor microenvironment (27, 36, 46, 53, 64, 70). Combining pulsed electric field therapy with immunotherapy is beneficial to mobilize the body’s immunity to kill tumors (37, 38, 66, 68). 5) It promotes systemic immunization and has the effect of distant effect, inhibiting tumors that may metastasize elsewhere (67, 70).

Although many studies of the effect of electric pulses on tumor microenvironment have been reported, there are still some questions that need to be addressed and more in-depth studies can be done in the future in the following areas.

1. The differences in the effects of IRE, nsPEF, and H-FIRE on cell and microenvironment need to be further studied. They have different parametric characteristics, the most prominent of which is the difference in pulse duration. They are capable of disrupting the structure of the cell membrane. However, nsPEF is characterized by high compression power, ultrashort pulse duration, fast rise time, and high electric field. When the pulse duration is shorter than the charging time of the cell membrane (mostly 100 ns), the charge cannot accumulate on the surface of the cell membrane and the applied electric field is mainly received by the membranes of intracellular organelles such as the nucleus, endoplasmic reticulum and mitochondria. When a 300 ns pulse (or longer) is applied, the pulse is long enough to allow the electric field to interact only with the plasma membrane and not the intracellular organelles (90, 91). The change of subcellular membrane potential may affect a series of signaling pathways. IRE and nsPEF are different in causing cell damage, which needs further study.
2. Even though it is the same modality, different parameters can bring about different changes. In IRE, the most studied is the voltage/field strength. Compared to a field strength of 500 V/cm, IRE using 2500 V/cm seems to be more capable of causing cellular damage, whether this is a thermal or non-thermal effect and by what exact mechanism of damage (including membrane damage, ATP depletion, mitochondrial damage, increase in ROS, DNA and protein damage) needs to be further investigated (4, 36). And changes in electric field strength bring about proportional changes in the mode of cell death, with the promotion of apoptosis evident at 500 V/cm but increased necrosis at 2500 V/cm (23), in between which there should be a suitable range of electric field strength that would keep the ablation zone within the desired range and cause more immunogenic death, but the appropriate field strength may vary with the conductivity of the ablated tissue changes.
3. The complex cascade of responses induced by IRE, nsPEF, and H-FIRE remains to be investigated. The effect of pulsed electric fields on Wnt/β-Catenin, KRAS, EGFR, and downstream NF-κB signaling may be critical in determining therapeutic strategies, as these signals are often dysregulated in tumorigenic development (92, 93). More studies should address the complex signaling cascade response activated after pulsed electric fields.
4. The structure of antigens released by pulsed electric fields is uncertain. In experiments *in vitro*, IRE, despite releasing the highest amount of protein, which could be due to membrane rupture, was present with 40% denatured proteins, possibly related to the interactions of the high electric field, the charged amino acid residues of proteins, and solvent molecules. Alterations in the secondary structure of proteins are essential for APC processing and antigen presentation (69). Future *in vivo* experiments are still needed to evaluate the antigenic characteristics of IRE or nsPEF release, which will be important to optimize its stimulation of APC and thus the initiation and activation of T cells.
5. The effect of IRE on microvasculature remains controversial. A study found that CD31 was increased at 7 days after IRE (1000 V; 100 ms; 1 Hz; 80 pulses) in the tumor area (46), but some studies found that CD31 was increased on day 4 after IRE (200 V/960 V, 100 us, 1 Hz, 20 pulses) but fell back at day 7 (36). The difference in parameters does not seem to explain this. What is certain, however, is that IRE does preserve local vascular structures better than other thermal ablations, and in the study by Bulvik et al. there was an observed infiltration of inflammatory cells around the vessels, which was not seen with radiofrequency ablation (73). Therefore, it is important to clarify whether IRE is able to create a time window with the right number of microvessels and increased permeability, as this could provide more support for the timing of combined immunotherapy.
6. The effect of IRE on immunomodulatory activity has become an area of intensive research. However, most previous studies have provided only some descriptive data on temporal level changes in immune cells. Less has been explored regarding the precise IRE-mediated immune response.
7. Energy-based local therapies and immunotherapy can be synergistically combined is also a future direction. Pulsed electric fields can promote antigen preservation and local inflammation, and synergistic effects exist between them and immunotherapy (37, 38, 49, 66–68).

## Conclusion

High voltage electrical pulses cause changes in multiple intracellular signaling pathways to inhibit replication and proliferation of tumor cells, and also kill tumor cells through multiple modes of death by necrosis, pyroptosis, and necroptosis. Pulsed electric fields can contribute to immunogenic death, increase tumor immunogenicity, reverse the immune tolerance environment, and can promote activation and proliferation of cancer-specific cytotoxic T cells acting locally and systemically.

## Author contributions

Study concept and design, YW. Acquisition of data, YW, LTX, HW, JZ, LX, CF, and TJ. Writing-Original draft preparation, YW. Visualization, YW. Obtaining of funding, TJ. Technical or material support, LTX, HW, and TJ. Study supervision, TJ. All authors contributed to the article and approved the submitted version.

## Funding

This study was supported by Development Project of National Major Scientific Research Instrument (82027803), National Natural Science Foundation of China (81971623), and Key Project of Natural Science Foundation of Zhejiang Province (LZ20H180001).

## Conflict of interest

The authors declare that the research was conducted in the absence of any commercial or financial relationships that could be construed as a potential conflict of interest.

## Publisher’s note

All claims expressed in this article are solely those of the authors and do not necessarily represent those of their affiliated organizations, or those of the publisher, the editors and the reviewers. Any product that may be evaluated in this article, or claim that may be made by its manufacturer, is not guaranteed or endorsed by the publisher.
